# Editorial: AI and genomics: crafting precision diagnostics in future pathology

**DOI:** 10.3389/fmed.2026.1918578

**Published:** 2026-07-20

**Authors:** Xin Qi, Balasubramaniam S

**Affiliations:** 1 Eisai Inc., Nutley, NJ, United States; 2 Digital University Kerala, Thiruvananthapuram, Kerala, India

**Keywords:** artificial intelligence, computational pathology, deep learning, genomics, machine learning, precision diagnostics, whole-slide imaging

Pathology is being reshaped by the convergence of artificial intelligence (AI) and genomics. A discipline long anchored in morphological interpretation is evolving into an integrative, data-driven science in which deep learning models, high-dimensional molecular profiles, and interpretable statistical methods together inform diagnosis, prognosis, and therapeutic selection. This Research Topic, “*AI and genomics: crafting precision diagnostics in future pathology*,” assembles 10 contributions that, taken together, illustrate how computational approaches are moving precision diagnostics from aspiration toward clinical reality. The articles span computational pathology of whole-slide images, machine-learning–based risk prediction, genomic biomarker discovery, genomically guided therapy, and the conceptual frameworks needed to integrate these layers responsibly.

A central theme is the coupling of tissue morphology with molecular endpoints through deep learning. Chen and Xu developed and compared three multiple-instance learning frameworks on routine hematoxylin and eosin whole-slide images to stratify 5-year breast cancer recurrence risk against the 21-gene recurrence score, with the strongest model reaching an AUC of 0.836, evidence that genomics-correlated risk stratification can be approximated directly from standard histology. In a complementary fusion of imaging and transcriptomics, Chen combined an optimized U-Net convolutional network for automatic quantification of bone erosion on joint CT with peripheral blood RNA sequencing in rheumatoid arthritis, linking imaging severity (correlated with DAS28) to RNA G-quadruplex-regulated inflammatory genes and offering a window onto post-transcriptional mechanisms of bone destruction.

Genomic biomarker discovery powered by machine learning forms a second thread. Zhang et al. integrated bioinformatics, LASSO and SVM-RFE feature selection, and *in vitro* validation to nominate EPYC, MAGED1, and LAP3 as diagnostic genes distinguishing rheumatoid arthritis from osteoarthritis (AUC > 0.85). Surveying the wider molecular landscape, Sousa et al. argue in their Opinion that whole-tumor transcriptome analysis, interpreted with machine-learning classifiers, captures functional tumor states invisible to static genomic sequencing, enabling finer diagnosis, prognosis, and therapeutic targeting in precision oncology. Ge broadens the lens further, reviewing how functional redundancy stabilizes microbial communities under antibiotic perturbation and highlighting AI-based tools for detecting latent resistance genes, a reminder that computational genomics extends to the microbial dimension of human disease.

The translation of molecular insight into therapy is exemplified by Wang and Yu, whose single-center analysis of trametinib as second-line treatment for KRAS G12C-mutant non-small-cell lung cancer reports meaningful disease control and identifies absence of bone metastasis and PD-L1 positivity as candidate response correlates an accessible, genotype-directed option where mutation-specific inhibitors are unavailable.

A further cluster demonstrates the value of interpretable machine learning for clinical risk prediction. Liu et al. built explainable ensemble models (best AUC 0.82) to predict impaired outcome in diabetic patients undergoing non-cardiac surgery, using Boruta and SHAP to surface vascular, renal, nutritional, and hemodynamic drivers of risk. Jiang et al. showed that dynamic C-reactive-protein trajectories, modeled by gradient boosting, accurately predict prolonged healing in diabetic wounds, with SHAP identifying the post-antibiotic CRP measurement as the dominant predictor. Dian et al. applied multifactorial regression to identify low body weight and elevated brain natriuretic peptide as independent predictors of post-intubation hypotension in hypertensive intracerebral hemorrhage, informing pre-emptive optimization. Complementing these quantitative models, Wu et al. grounded diagnostic precision in careful clinical observation, demonstrating through a comparative dermoscopy study that distinct vascular and structural features differentiate extramammary Paget's disease from chronic eczema and support earlier, more accurate diagnosis.

Read collectively, these contributions sketch both the promise and the unfinished agenda of AI-enabled, genomics-informed pathology. Several recurring lessons emerge. Interpretability is no longer optional: SHAP analysis, transparent feature selection, and explainable architectures appear across the studies as prerequisites for clinical trust. Integration is the frontier, whether linking whole-slide images to recurrence scores, CT phenotypes to transcriptomic regulation, or molecular subtype to drug response. Yet, most models remain single-center and retrospective, and the authors themselves rightly emphasize external validation, calibration, standardized pipelines, and prospective testing before deployment. Bridging the gap between strong experimental performance and regulatory-grade clinical tools, as several contributors note, is the defining challenge ahead.

I thank all of the authors for their contributions, the reviewers for their careful evaluation, and the Frontiers editorial team for their support in bringing this Research Topic to completion. I hope these articles encourage continued collaboration among computational scientists, pathologists, geneticists, and clinicians as the field works to translate AI and genomics into diagnostics and ultimately therapies tailored to the individual patient.

